# Catalytic activity evaluation of phyto synthesized SrO and chitosan encapsulated SrO nanomaterials

**DOI:** 10.1371/journal.pone.0328646

**Published:** 2025-08-13

**Authors:** Maria Zaib, Kaif Akram, Tayyaba Shahzadi, Indra Neel Pulidindi, Awais Khalid, Mousa M. Hossin, Hanadi A. Almukhlifi, Ayesha Siddiqua, M. Yasmin Begum

**Affiliations:** 1 Department of Chemistry, University of Jhang, Jhang, Pakistan; 2 Department of Chemistry, Government College Women University Sialkot, Sialkot, Pakistan; 3 School of Science, GSFC University, Vadodara, India; 4 Department of Physics, College of Science and Humanities in Al-Kharj, Prince Sattam bin Abdulaziz University, Al-Kharj, Saudi Arabia; 5 Chemistry Department, Faculty of Science, Albaha University, Albaha, Saudi Arabia; 6 Department of Chemistry, Faculty of Science, University of Tabuk, Tabuk, Saudi Arabia; 7 Department of Clinical Pharmacy, College of Pharmacy, King Khalid University, Abha, Saudi Arabia; 8 Department of Pharmaceutics, College of Pharmacy, King Khalid University, Abha, Saudi Arabia; Siksha O Anusandhan University Institute of Technical Education and Research, INDIA

## Abstract

Industrial wastewater contaminated with organic dyes poses significant environmental and health risks. Developing effective and eco-friendly removal methods is crucial to mitigate the harmful impact of dye pollution. This study explores the green synthesis and application of strontium oxide (SrO) nanoparticles and chitosan-encapsulated strontium oxide (CS-SrO) nanocomposite as adsorbents for the removal of cationic and anionic dyes from aqueous solutions. Characterization revealed that the SrO nanoparticles exhibited a sharp localized surface plasmon resonance (LSPR) peak at 274 nm, while the CS-SrO nanocomposite depicted a broad LSPR peak between 230–280 nm. XRD analysis confirmed the face-centered cubic structure of the SrO nanoparticles, with an average crystallite size of 32 nm. Detailed dye removal studies revealed that the SrO nanoparticles achieved up to 80% removal of the cationic crystal violet dye, while the CS-SrO nanocomposite demonstrated 81% removal of the anionic methyl orange dye under optimized conditions. Further investigations into adsorption kinetics, isotherms, and thermodynamics provided insights into the mechanisms governing the dye removal processes. This work contributes to the advancement of green nanotechnology for environmental remediation purposes. These findings pave the way for the development of practical applications in water purification and pollution control strategies.

## Introduction

Water contamination caused by organic pollutants has emerged as a grave concern in recent years. There are several underlying factors contributing towards this contamination, but the primary cause that seriously worsens the quality and condition of the ecosystem are organic dyes. Dyes are extensively used throughout small and large-scale industries, including tanneries, culinary, cosmetic, textile, and pharmaceutical sectors, with a net production of one million tons worldwide [1].

Dyes are synthetic materials and have complicated aromatic structures, which ensure their stability [2]. In addition to degrading the appearance of natural water bodies, the hazardous nature of dyes damages the flora and fauna of the natural environment. Dyes reduce light penetration, thus they can affect aquatic life behavior during photosynthetic processes [3]. In addition, some dyes are poisonous and can cause cancer in a variety of aquatic organisms. Additionally, these dyes have been established as human health threats due to the development of various diseases, syndromes, and disorders [4]. It is, therefore, necessary to introduce effective techniques that can remove dyes from wastewater streams.

Several techniques have been reported that can remove dyes from wastewater sources. Ion exchange, biological treatment, coagulation and flocculation, electrochemical treatment, advanced oxygen processes (AOP), and membrane filtration are some of the significant ways mostly employed for dye removal strategies. None of these techniques could be tended to as the best accessible technology since every method enjoys its benefits and restrictions related to cost, effectiveness, achievability, and ecological effects [5]. Among all these methods, adsorption proved itself as a promising tool due to cost effectiveness, high design flexibility, efficiency to remove small pollutant molecules, resistance to toxic matter and easy handling [6]. The concept of adsorption has been completely revolutionized by the introduction of nanomaterials. Extensive surface area, ease to modify superficial and electron conduction characteristics, facilitates their application as an adsorptive material [7].

The deacetylated derivative of chitin is known as poly-(1–4)-2-amino-2-deoxy-d-glucose or chitosan. Chitosan has found extensive applications as an adsorbent due to its low price and non-toxic nature. The adsorbent capacity of chitosan is very high, i.e.,1000–1100 g/kg. It can efficiently remove pollutants and is ranked above activated carbon. The capacity of chitosan to bond with pollutants via surface NH_2_ and OH groups is the reason of this high removal capacity [8]. Additionally, it has been discovered that this efficiency can be significantly enhanced when they are combined with metal oxide nanoparticles to form composites [9]. For instance, immobilized magnesium oxide nanoparticles on chitosan surface were discovered to be a superior material removing methyl orange dye [10].

**Table:** 

Adsorbent Material	Dye Removal Efficiency (Max %)	Target Dyes	Synthesis Cost	Environmental Impact	Ref
SrO nanoparticles	80% (cationic CV)	Crystal Violet	Low*	Green synthesis reduces chemical waste	
CS-SrO nanocomposite	75-80% (anionic MO)	Methyl Orange	Low*	Biodegradable chitosan reduces toxicity	
Activated Carbon	60-95% depends on modification	Multiple dyes	High	High economic cost, non-renewable	[11,12]
Biochar	40-85%	Various contaminants	Low	Carbon-negative footprint, residual waste-derived	[13]
Clay Minerals	50-75%	Cationic dyes	Very Low	Abundant but requires modification	[14]
Metal-Organic Frameworks	85-99%	Specific dye targets	Very High	Synthetic chemicals may cause secondary pollution	[15]

In this study, strontium oxide nanoparticles and their chitosan composite were prepared using fresh leaves of *Melia azedarach*. Then both prepared materials were comparatively employed for removal studies of a cationic (crystal violet) and anionic (methyl orange) dye. Here a green and environmentally friendly method for creating nontoxic, biodegradable nanomaterials is chosen [16,17]. Plant biodiversity has received considerable attention as it synthesizes metal as well as metal oxide nanoparticles due to the occurrence of potent phyto constituents. Chinaberry, or *Melia azedarach*, is a member of the Meliaceae family. *M*. *azedarach* is known to exhibit antibacterial, antipyretic, diuretic, and bug repellant properties. Key phytochemicals of *Melia azedarach*’s are ferulic acid, catechin, rutin, myricetin, meliartenin and anthraquinone [18]. As per our knowledge, no previous study has been reported in this perspective.

## Materials and methodology

### Reagents

All the chemicals (Sr(NO_3_)_2_, chitosan_,_ CH_3_COOH_,_ NaOH_,_ NaNO_3_, Na_2_CO_3_, Na_2_SO_4_, NaCl, crystal violet (95%, Sigma-Aldrich), methyl orange (85%, Merck) used in this study were of analytical grade and used as received. Solutions were prepared with distilled water on daily basis.

### Preparation of leaf extract

Leaves of *Melia azedarach* were collected from Government College Women University Sialkot (32.5° N, 74.5° E). Collected leaves were washed with distilled water and shade dried for 7–10 days. Afterward, they were grinded into fine powder. About 5 g of powder was heated in 100 mL of distilled water at 75 ºC-80 ºC for 1h. Then, the leaf extract was collected by filtration using Whatman filter paper no 1. Prepared leaf extract was stored at 4 °C [19].

### Preparation of strontium oxide nanoparticles (SrO)

Leaf extract and strontium salt solution of definite concentration was mixed and stirred in a volume proportion of 5:1. Later, it was left for 24 h at room temperature. After the recommended time period, obvious color change can be observed. In the end, NPs were collected by centrifugation. During centrifugation, NPs were washed with distilled water and ethanol on alternate basis. Collected nanoparticles were dried in hot air oven [20].

### Preparation of chitosan strontium oxide nanocomposite (CS-SrO)

Definite amount of synthesized SrO nanoparticles (3 mg) was dispersed in distilled water. A specific amount of chitosan (0.25 g) was mixed in 1% CH_3_COOH by continuous stirring for 1 h. Dispersed NPs were mixed with chitosan solution and stirred for 10 min. Then the sol was ultra-sonicated for 2 h. The suspension was poured into petri dishes and dried in the oven at 35 ºC for 24 h leading to solid film formation. Finally, the nanocomposite was stored in an airtight container [21].

### Dye removal studies

Prepared SrO NPs and CS-SrO nanocomposites were employed for adsorption studies of crystal violet and methyl orange dye. Percentage of removal depends on various factors, i.e., dosage of prepared nanoparticles and nanocomposite (5–100 mg), pH of dye solution (2–10), contact time (30–180 min), initial dye concentration (5–100 mg/L) and interfering species. These factors were studied in detail in order to determine optimized conditions. Results were recorded by using UV-Vis spectrophotometer (Specord 210 plus Analytic Jena, Gottingen, Germany) at λ_max_ value of 590 nm and 462 nm for crystal violet (CV) and methyl orange (MO) dyes respectively. Following equation was used to determine percentage removal values:


$$\;\%\;Removal\;of\;Dyes=\;\frac{A_{i}-A_{f}}{A_{f}}\times 100$$ (1)

where A_i_ is initial absorbance of dye before adding nanocatalyst, A_f_ is final absorbance of dye after adding nanocatalyst.

### Determination of point zero charge

The point zero charge of CS-SrO nanocomposite and SrO NPs was calculated by using the salt addition method. The overall surface charge on the material surface is zero at pH_pzc_. Point zero charge was calculated using batch equilibrium technique. A 40 mL solution of NaNO_3_ (0.1M) with a pH range of 2–10 was added to a number of flasks along with 5 mg of the prepared material [22].

Each bottle was closed and shaken for 48 hours at normal temperature to modify initial pH. Final pH of the solution was then determined using a pH meter. ΔpH was used to calculate the total charge adsorbed (the difference in the value of initial pH and after 48 hr). Extrapolation of attained curve with pH initial axis showed pH_pzc_ value.

### Characterization

UV-Visible spectra were noted between the range of 150 nm to 400 nm with spectrometer (Specord 210 plus Analytic Jena, Gottingen, Germany). FTIR spectra were recorded in the range of 4000 cm^-1^ to 500 cm^-1^ by using Nicolet 6700 FTIR spectrophotometer (Thermo Fischer Scientific, US). XRD spectra analysis was carried out with X ray powder diffractometer (JEOL JDX −3623, Japan) in the range of 10°- 80° with CuK_α_ radiation having 0.15406 nm wavelength. SEM with EDX analysis (Tescan Vega3, Czechia) was performed at scale 5 µm, 2 µm, 1 µm and 500 nm with magnification of 5000X, 10,000X, 25,000X and 50,000X.

## Results and discussion

### Characterization Studies

#### UV-Visible spectroscopic analysis.

**Fig 1:**
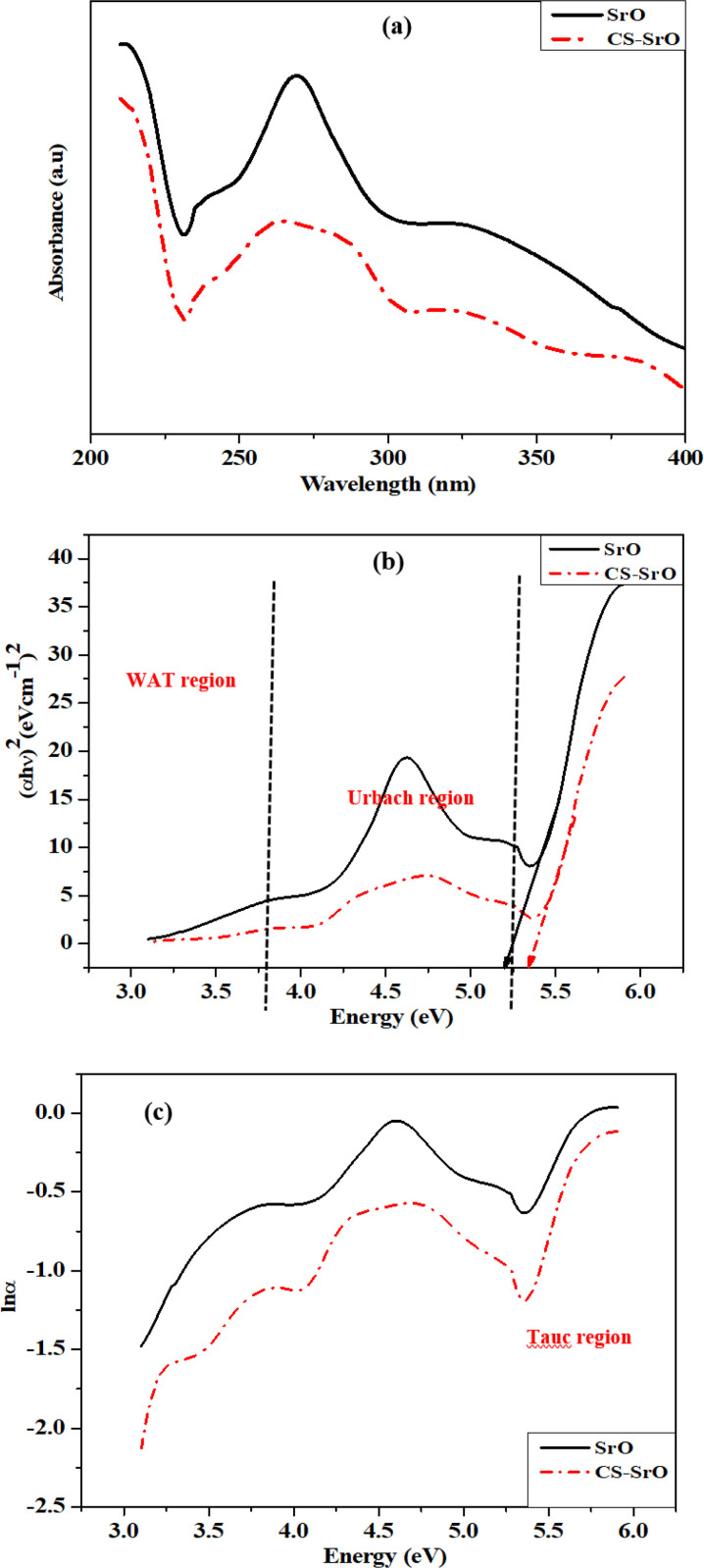
(A) UV-Vis spectra of SrO NPs and CS-SrO nanocomposite (B) Tauc plots of SrO and CS-SrO (C) Urbach energy diagrams of SrO and CS-SrO.

Similar patterns of absorption profiles with minor differences in the maxima values can be seen in the UV-Vis spectra Fig 1A of CS-SrO nanocomposite and SrO NPs. Due to the lack of conjugated double bonds in chitosan, the UV-Vis spectrum of chitosan (not shown here) did not exhibit any distinctive absorption peak [23]. SrO NPs shows a distinct peak around 267 nm. This value closely resembled another study data in which SrO nanoparticles were prepared from *Ocimum sanctum* leaf extract with λ_max_ value of 274 nm. This reported peak is due to quantum size effect of monodispersed colloidal particles [24]. However, the UV-Vis spectrum of CS-SrO nanocomposite has a broad peak between 230–280 nm. This is due to interaction of SrO NPs with chitosan.

Band gap energy values were calculated from Tauc plots using following equation (2):


$$\left(\alpha hv{)}^{n}=k(hv-Eg\right)$$ (2)

where α is absorption co-efficient, n is index or optical transition of semiconductors, K is proportionality constant depends on material, hʋ is photon energy in eV and Eg is band gap energy.

With reference to Fig 1B, band gap values for SrO and CS-SrO is found to be 5.21 and 5.34 eV. This blue shift can be explained on the basis that composite synthesis not only favors the elemental energy level formation but also affect the intrinsic energy levels. This is due to hybridization of orbitals between the strontium oxide and chitosan. As the crystallite size is much larger (calculated later under XRD data analysis discussion) than the Bohr excitation radius thus quantum confinement effect does not impact the band energy value. Similar findings are reported in literature [25,26]

Urbach energy Fig 1C is calculated by using following equation (3) and taking slope reciprocal from plot drawn between ln α and hυ


$$\alpha =\alpha_{0}+\exp(\frac{E}{Eu})$$ (3)

Urbach energy represents the localized defect states and usually correlated with the disorder present in framework of fabricated material. As per reported studies, band gap energy and Urbach energy exhibit an inverse relation. Similarly in this study, CS-SrO (0.49 eV) demonstrates a lower Urbach energy value than SrO (0.76 eV) [27].

#### Fourier transform infrared (FTIR) spectroscopic analysis.

**Fig 2:**
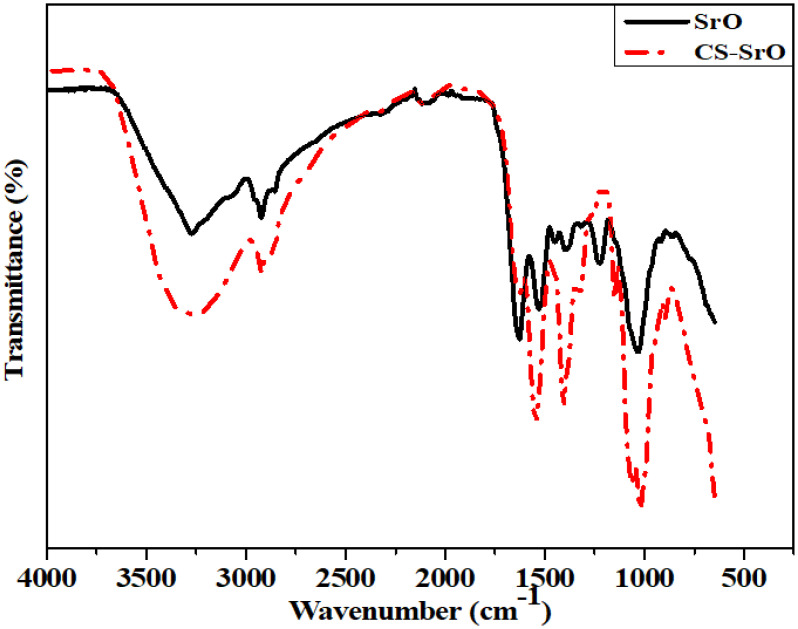
FTIR spectra of green synthesized SrO and CS-SrO.

The FTIR spectrum of green synthesized SrO NPs is displayed in Fig 2 within the range of 4000–750 cm^-1^. Notable peaks in biosynthesized SrO nanoparticles can be seen at 3280 cm^-1^, 2922 cm^-1^, 2117 cm^-1^, 1625 cm^-1^, and 1446 cm^-1^ respectively. The stretching vibrations of the OH (hydroxyl) groups are responsible for the IR peak at 3280 cm^-1^ [28]. Characteristic peak at 1446 cm^-1^ might be attributable to the asymmetric stretching vibration of the strontium oxide (Sr-O) band, while the transmittance value at 1625 cm^-1^ matches the stretching vibration of carbonyl (C-O) groups [29]. Finally, the carbon-hydrogen (C-H) group stretching vibration is reflected in the peak of 2922 cm^-1^. This result supported the idea that the leaf extract of *Melia azedarach* could play the role of a reducing and capping agent.

Main functional groups of CS-SrO nanocomposite are shown in Fig 2. The peak at 3272 cm^-1^ shows OH- groups while the peak at 2922 cm^-1^ depicts C-H bond of CH_3_ groups. Peaks at 1625 cm^-1^ and 1379 cm^-1^ represent stretching vibrations of the carbonyl group (R-CO-NH_2_) and bending vibrations of the CH_2_ groups [30]. The bands at 1148 and 1029 cm^-1^ relate to C-O-C symmetric and asymmetric stretching vibrations [31]. All these bands are taken into account as proof that the chitosan structural features were preserved even after the SrO nanoparticles were incorporated into the polymer matrix. These results confirm that the chitosan phase serves as a matrix for SrO nanoparticles assembling and indicate that some intermolecular interactions may occur between chitosan and SrO in the composite [32].

#### X-ray diffraction analysis.

**Fig 3:**
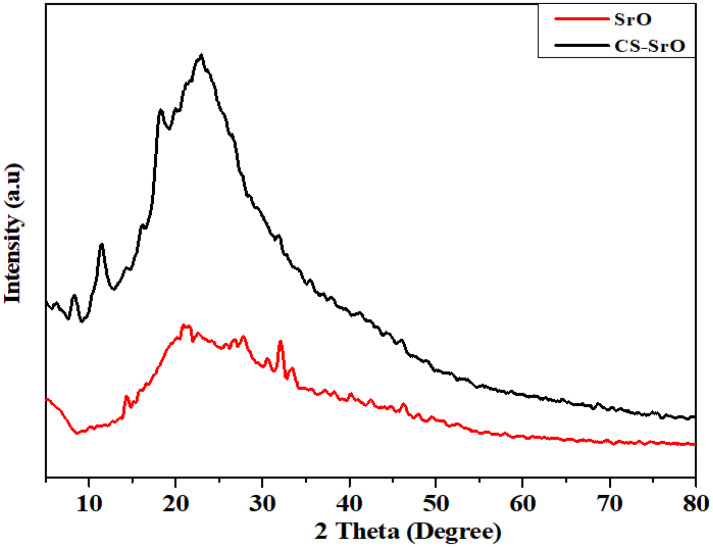
X-ray diffraction spectra of green synthesized SrO NPs and chitosan strontium oxide nanocomposite.

Fig 3 compares the XRD pattern of the SrO NPs and CS-SrO nanocomposite. The diffraction intensity was recorded from 10˚ to 80˚.

X-ray diffraction pattern of SrO NPs shows prominent peaks at 2θ values of 32˚ and 46.5˚. These peaks correspond to crystal plane (110) and (200) for fcc SrO with reference to JCPDS 48–1477 [33]. Debye-Scherrer formula is used to calculate the average crystallite size of strontium oxide nanoparticles:


$$D=\frac{K\lambda }{\beta cos\theta }$$ (4)

Where λ is the wavelength of X-ray (0.15406 nm), β is full width at half maximum, K is the Scherrer constant (0.9) and θ is the Bragg angle. The average crystallite size for SrO nanoparticles is calculated to be 32 nm.

XRD pattern of CS-SrO shows peaks at 10˚ and 23˚ illustrating semi semi-crystalline pattern of chitosan following JCPDS file no 039–1894 [34]. Other characteristics peak at 32˚, 45.9˚ and 51˚ corresponds to crystal plane values (110), (200) and (220) for face centered cubic structure of SrO [33]. According to these results, the CS-SrO composite is successfully synthesized by dispersing SrO NPs into the CS matrix [32]. The average crystallite size for CS-SrO NPs is found to be 26 nm.

#### Scanning electron microscopy (SEM).

**Fig 4:**
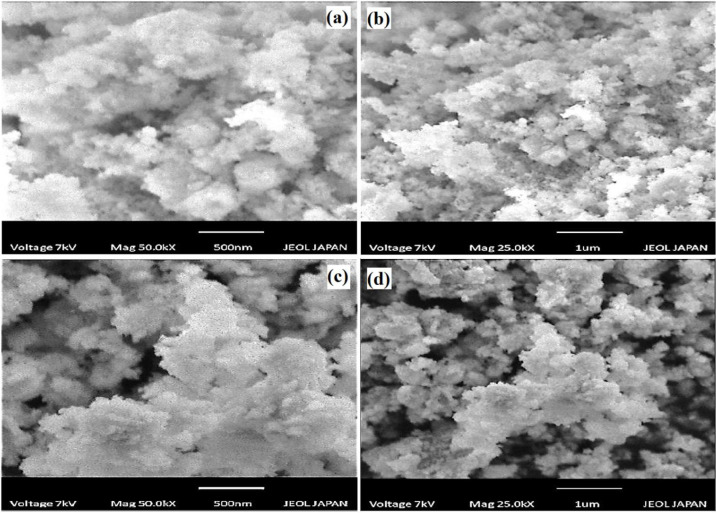
SEM images of SrO nanoparticles at the scale of (A) 500nm (B) 1µm, and CS-SrO nanocomposite at the scale of (C) 500nm (D) 1µm.

Fig 4A and 4B displays the morphology of synthesized strontium NPs. Synthesized SrO NPs illustrate a smooth and almost spherical surface. Individual nanoparticles aggregate to form clusters [35]. Fig 4C and 4D shows the morphology of CS-SrO. The SrO NPs appeared as white dots that were uniformly dispersed across the polymer matrix’s surface. However, the jumbled surface is caused by the polymer layer’s adsorption onto the particle surface [36].

#### Energy dispersive X-ray spectroscopic analysis.

**Fig 5:**
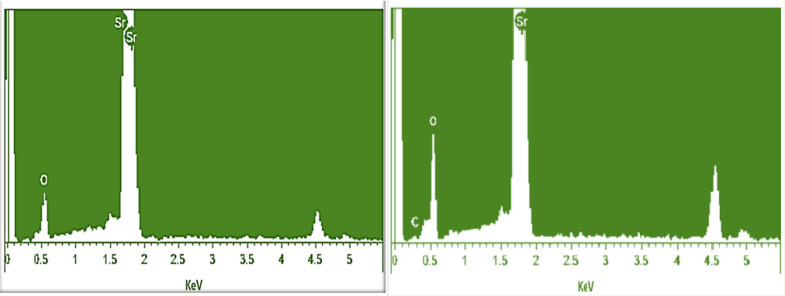
EDX spectra of green synthesized (A) SrO NPs and (B) CS-SrO.

Fig 5A shows the elemental composition of SrO NPs that was determined by energy dispersive X-ray spectroscopy. Internal energies between 0 and 6 keV were used for EDX analysis. At 2 keV, strontium displayed a typical adsorption peak [20]. The two peaks of Sr correspond to K_α_ and K_β_ emission. Strontium and oxygen element content percentages are 85.02% and 14.98% respectively. The EDX examination demonstrates that the strontium oxide nanoparticles are entirely composed of Sr and O, depicting sample’s purity [24].

Elemental composition of chitosan-SrO nanocomposite was evaluated with EDX graph displayed in Fig 5B. It demonstrates peaks for C (10.20%), Sr (80.00%) and O (9.70%). The presence of strontium peak with the major percentage content value indicates that it had been incorporated into the polymer [36].

#### Point zero charge.

**Fig 6:**
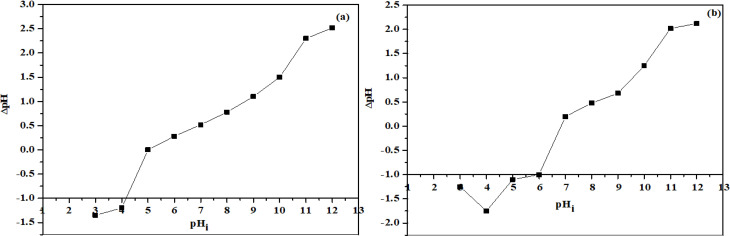
Point zero charge of(A) SrO nanoparticles and (B) CS-SrO nanocomposite.

Nature of binding sites on the prepared material can be determined by pH_pzc_. Point of zero charge is important for the determination of charge on the surface of under study nanoparticles and nanocomposite [37]. Adsorption of positively charged ions is favored by the pH > pH_pzc_ and vice versa for negatively charged ions. According to the results depicted in Fig 6A and 6B, the point of zero charge for the CS-SrO nanocomposite and SrO NPs is 6.8 and 5 respectively. These results showed that below these pH values, the surface becomes positive and attach oppositely charged anions by electrostatic interaction. While with pH conditions more basic than the pH_pzc_, nanomaterials prefer to bind basic or cationic dyes [38].

### Dye removal studies

#### Effect of dosage.

**Fig 7:**
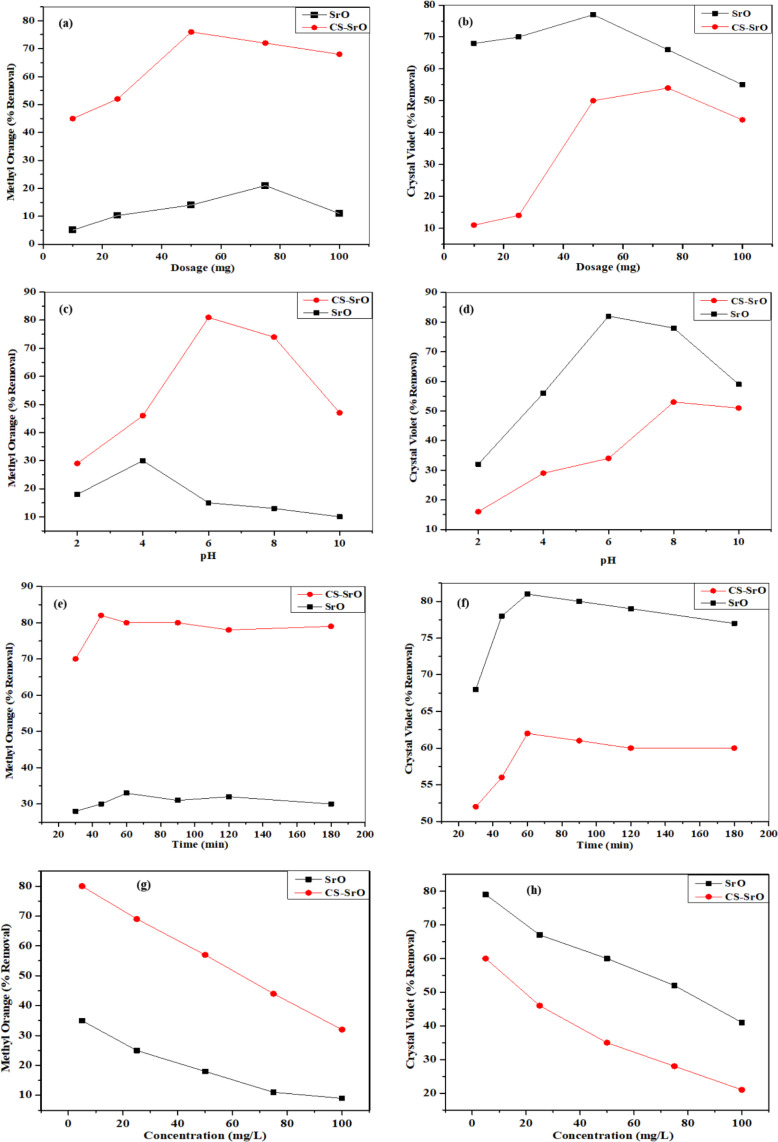
Comparative graph showing effect of nanocatalyst dosage on removal of dyes by SrO nanoparticles and CS-SrO nanocomposite (C dye = 5 mg/L, V = 25mL, Time = 120 min). (A) methyl orange, (B) crystal violet, (C) effect of pH on the removal of methyl orange (dosage = CS-SrO (50 mg), SrO (75 mg)), (D) effect of pH on crystal violet dye, (E) effect of contact time on removal of methyl orange (pH = 6), (F) effect of contact time on removal of crystal violet (pH = 8), (G) effect of dye concentration on removal of methyl orange (V = 25 mL, Time = 60 min (SrO), 45 min (CS-SrO), pH = 6 (CS-SrO) 4 (SrO) and dosage = CS-SrO (50 mg), SrO (75 mg)), and (H) effect of dye concentration on removal of crystal violet (V = 25mL, Time = 90 min (SrO), 60 min (CS-SrO), pH = 8 (CS-SrO), 6 (SrO) and dosage = CS-SrO (75 mg), SrO (50 mg)).

The effect of nanocatalyst concentration is studied by maintaining all the other experimental conditions constant (C_dye_ = 5 mg/L, V = 25 mL) while varying the nanocatalyst dosage of SrO NPs and CS-SrO nanocomposite from 5 mg to 100 mg respectively, results are presented in the Fig 7A and 7B.

Both the graphs depicted that amount of NPs and composite depicted a linear increasing trend up to a certain point. Afterwards, further increase in dosage would not enhance the percentage removal. In this scenario, the agglomeration of the particles could reduce dye removal efficiency; as a result, the effective surface area reduces and a decrease in efficiency is observed [39]. In the case of CS-SrO nanocomposite, dose of 50 mg achieved the maximum dye removal percentage of 76%, which can be associated with a higher amount of adsorption sites [10]. Thus, 50 mg of CS-SrO composite is considered to be the optimum dosage. Whereas the maximum % removal of dye by SrO NPs is 21% for 75 mg dosage. Acidic dyes generate negatively charged species in aqueous solution and provide basic medium that imparts negative charge to nanoparticles. Further -OH group of nanoparticles is deprotonated more easily in comparison to hydrogen bonded hydroxyl and amide groups of composites. As SrO NPs are negatively charged due to which very small percentage of anionic dye is removed.

In case of cationic dye, the optimum dosage of SrO NPs is 50 mg with 77% removal percentage whereas the optimum dosage of chitosan-strontium oxide nanocomposite is 75 mg with 54% dye removal efficiency. Less removal of cationic dye by nanocomposite from solution is credited to positive surface charge of chitosan due to protonation of the –NH_2_ groups [20,40].

#### Effect of pH.

Another crucial factor that influences the removal of dye is the solution pH. By maintaining a constant concentration of dye (5 mg/L), dosage (CS-SrO nanocomposite-50 mg and SrO NPs 75 mg), and contact time (60 min), influence of dye pH on the removal studies is observed. The experiment is conducted under pH conditions varying from 2 to 10 by using 0.1 M HCl or 0.1 M NaOH.

According to Fig 7C, in case of methyl orange, the optimum dye removal percentage is attained at pH 6 and 4 for CS-SrO nanocomposite and SrO NPs respectively. Effect of pH on dye removal can be described by point zero charge, or pH_PZC_. pH_PZC_ value for nanocomposite and NPs is 6.8 and 5 respectively. So, at pH < pH_PZC_ the overall charge on the surface of material is positive due to adsorption of extra H^+^ ions [41]. In both cases, favorable interaction is observed at pH values lower than their point zero charge. As at these pH values, both acquire positive surface charge that allows the electrostatic attraction against the oppositely charged dye molecules and facilitates removal of dye molecules [42]. However, as pH increases, there is a reduction in the efficacy of the dye removal process, which may have been caused by hydroxyl ions competition with the dye molecules for binding sites. Thus, it is evident that 6 and 4 is the optimum pH value for dye removal studies by nanocomposite and NPs respectively [43].

Similarly effect of pH for the removal of crystal violet dye can be discussed on the basis of point zero charge Fig 7D. Here in this case, the values of optimum pH for CS-SrO nanocomposite and SrO NPs are 8 and 6 respectively. Both these values are greater than point zero charge values. As we know that pH > pH_PZC_ favors formation of negative surface sites. Hence this situation eases the interaction of cationic dye with oppositely charged binding sites. However, under highly basic conditions, excess -OH molecules competing with the nanomaterials hampered removal efficiency for CV dye.

#### Effect of contact time.

Adsorption studies of methyl orange and crystal violet dye Fig 7E and 7F is investigated for different time intervals (30–180 min) with 25 mL of initial dye solution (5 mg/L) under optimum pH and dosage values respectively. Initially, in both cases, percentage of dye removal is enhanced by increasing the contact time. After a certain time limit, saturation is observed to be attained indicating maximum interaction. In case of methyl orange, 45 min (composite) and 60 min (NPs) was required for attainment of equilibrium situation. While in case of crystal violet dye, 60 min (composite) and 90 min (nanoparticles) was recorded.

#### Effect of initial dye concentration.

The effect of initial dye concentration (C_dye _= 5 mg/L, 25 mg/L, 50 mg/L, 75 mg/L, 10 0 mg/L) is observed at fixed dosage and pH values for nanoparticles and nanocomposites respectively.

It can be seen Fig 7G and 7H that the dye’s removal percentage has decreased despite the increase in initial concentration. Lower dye concentration value, along with an excess of available binding sites, speed up the dye removal process initially. The saturation of accumulation sites lower removal percentage at higher concentrations. Thus, the percentage of dye removal is higher at smaller initial values and vice versa. This clearly shows that the initial concentration of MO or CV affects its ability to be adsorbed from aqueous solution [44].

After optimizing several factors for dye removal studies, SrO nanoparticles are considered as an effective nanomaterial for crystal violet removal owing to their negatively charged surface. It depicts maximum interaction with cationic dye, resulting in 80% removal percentage value from aqueous solution in comparison to its nanocomposite. Similar results have been reported in literature [20]. Whereas, for removal of anionic dye (MO), CS-SrO nanocomposite has depicted better results with 81% dye removal percentage. Table 1 summarizes the comparative analysis of SrO NPs and CS-SrO nanocomposite for removal of crystal violet and methyl orange dyes from aqueous medium.

**Table 1:** Comparative analysis for adsorption of MO and CV on CS-SrO nanocomposite and SrO nanoparticles.

Materials	Dye	Optimum pH	Optimum dosage	Time	% Removal
SrO nanoparticles	Methyl orange	4	75 mg	60 min	29%
CS-SrO nanocomposite	Methyl orange	6	50 mg	45 min	81%
SrO nanoparticles	Crystal violet	6	50 mg	60 min	80%
CS-SrO nanocomposite	Crystal violet	8	75 mg	90 min	58%

### Interference studies

Wastewater streams contain different types of salts, and their presence definitely affects the adsorption capacity. Adsorption of methyl orange dye by biopolymeric composite seems to be more affected as the anionic parts of different salts offer appreciable hindrance to the attachment of dye molecules with the positively charged surface of the nanocomposite. Further in this case, chloride, sulphate, and carbonate seem to decrease the percentage removal value to a greater extent than nitrate. This illustrates more interaction between the first three due to higher negative charge value (sulphate and carbonate) or greater charge density value of chloride ion.

**Fig 8:**
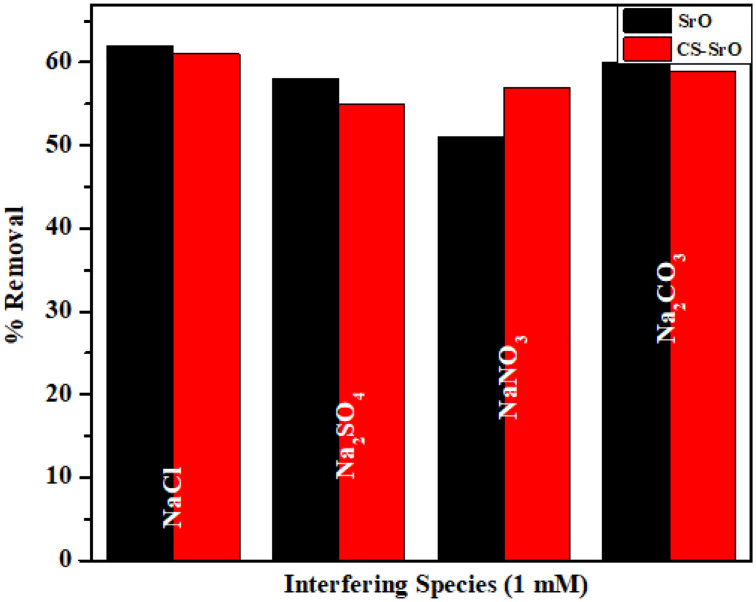
Comparative graph showing effect of interfering species on the removal of crystal violet by SrO and methyl orange by CS-SrO.

In case of NPs, except nitrate all other interfering species does not appreciably decrease the removal efficiency (Fig 8).

### Regeneration studies

In regeneration studies, respective loaded nanomaterial was dispersed in 25 mL of absolute ethanol for a definite time period. Then it was filtered, washed several times with distilled water and dried for next adsorption cycle. The adsorption-desorption cycle was repeated thrice to evaluate the regeneration performance.

**Fig 9:**
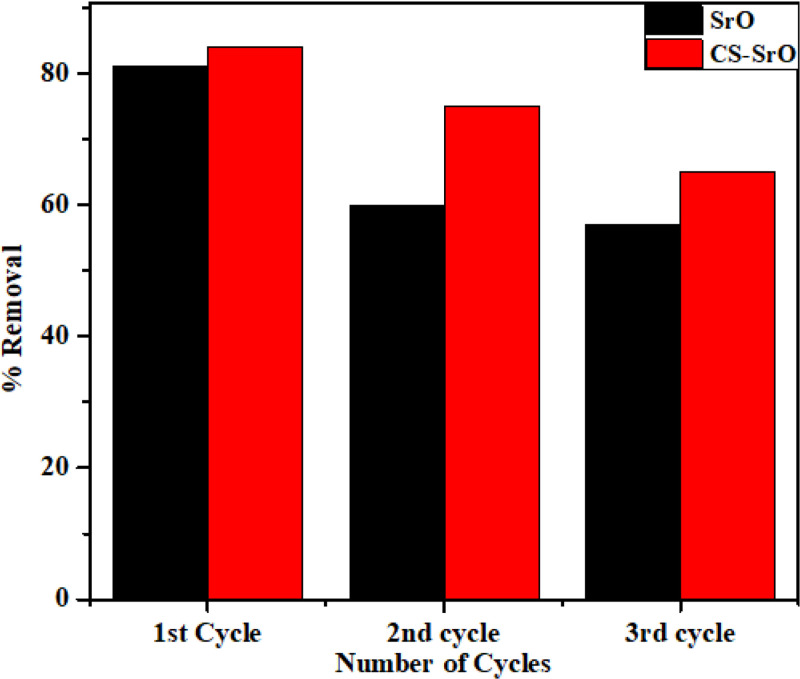
Regeneration studies for the removal of crystal violet by SrO and methyl orange byCS-SrO.

The bar chart Fig 9 shows that in comparison to the NPs, the regenerating ability of the composite was better. However, the impact of adsorbed molecules on the active sites may be the cause of the gradual decline in efficiency with the increased number of repeated cycles.

### Dye removal mechanism

Heat energy generated during the interaction of respective nanocatalyst/dye system stimulated the transfer of electrons from valence to conduction band. This facilitates formation of holes in valence band. Ultimately these electrons and holes react with oxygen and water molecules leading to formation of superoxide and hydroxyl radicals.

**Fig 10:**
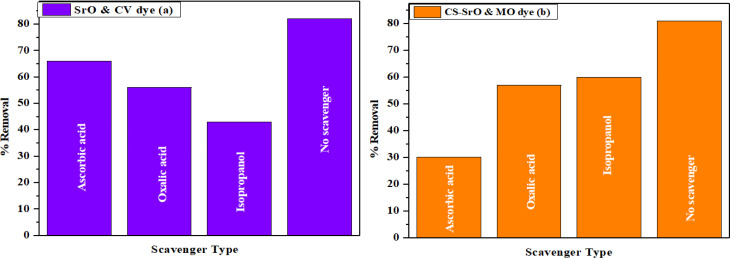
Scavenging radical experiments for(A) SrO/CV and (B) CS-SrO/MO.

Radical scavenging experiment was carried out to demonstrate the impact of actual reactive species. In this experiment, oxalic acid (OA), ascorbic acid (AA) and iso propanol (IP) were taken as quencher for holes, hydroxyl and superoxide radicals. Effect of these scavengers on dye removal studies is depicted in Fig 10A and 10B. In case of SrO NPs and CV dye, 82% percentage removal is observed in the absence of any scavenger. This value is decreased to 66, 56 and 43% in presence of AA, OA and IP. On the other hand, for the CS-SrO nanocomposite and MO dye system, value of 81% in the absence of any scavenger is noted. This removal efficiency is decreased to 30, 57 and 60% in presence of AA, OA and IP.

Thus, it can be concluded that superoxide and hydroxyl radicals played a crucial role in dye removal studies of CV and MO dye by SrO and CS-SrO respectively.

### Adsorption isotherm

Here the adsorption isotherms are discussed as the adsorption is considered to be an important step of dye removal studies by nanocatalyst.

Langmuir and Freundlich models were considered in the current work to investigate the MO and CV removal by CS-SrO nanocomposite and SrO NPs. Only these cases were considered as they showed maximal interactive phenomena. Equation no 5 and 6 illustrated the linear version of the Langmuir and Freundlich models.


$$\frac{1}{q_{e}}=\;\frac{1}{q_{m}K_{L}C_{e}}\;+\frac{1}{q_{m}}$$ (5)

$$\log q_{e}=\;logK_{f}+\frac{1}{n}\log C_{e}$$ (6)

**Fig 11:**
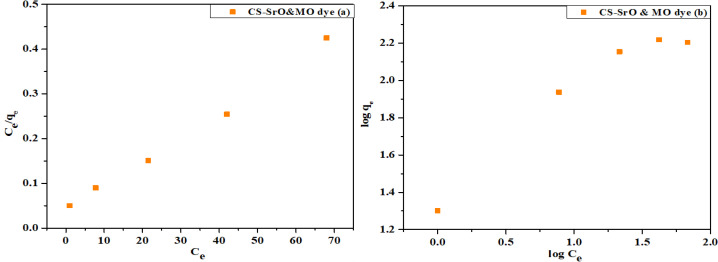
Isotherm models for adsorption of MO dye on CS-SrO nanocomposite (A) Langmuir model and (B) Freundlich model.

**Fig 12:**
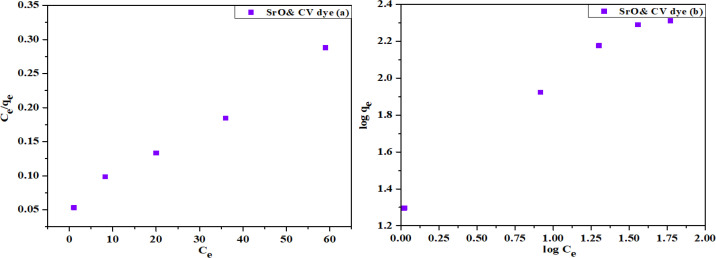
Isotherm models for adsorption of CV dye on SrO nanoparticles (A) Langmuir model, and (B) Freundlich model.

Where q_e_ is amount of dye adsorbed at equilibrium, C_e_ is the equilibrium concentration of dye in solution, q_m_ is maximum adsorption capacity, and K_L_ is the Langmuir constant. Values of q_m_ and K_L_ were obtained from slope and intercept of plot shown in Fig 11A and Fig 12A. In equation 4, K_f_ is Freundlich constants representing adsorption capacity, and 1/n is the adsorption strength. Values of 1/n and K_f_ derived from slope and intercept of a graph depicted in Fig 11B and Fig 12B. Langmuir model described monolayer adsorption at homogeneous sites having same energy whereas Freundlich model described adsorption on a heterogeneous surface having different energy sites.

Calculated isotherm parameters shown in Table 2 indicate the value of R^2^ (correlation coefficient) for both models was > 0.9 but MO and CV adsorption on CS-SrO and SrO respectively is better explained by Langmuir model because the values of R^2^ for Langmuir is greater than Freundlich model. R_L_ value for the MO and CV adsorption onto CS-SrO nanocomposite and SrO nanoparticles were determined to be 0.1 and 0.2 respectively this is less than 1 which supports favorable adsorption process.

**Table 2:** Parameters of isotherm models for adsorption of MO and CV on CS-SrO and SrO respectively.

Material	Dye	Langmuir model				Freundlich model		
		Exp. (mg/g)	(L/mg)			1/n	(mg/g)	
**CS-SrO** **nanocomposite** **SrO** **Nanoparticles**	**MO** **CV**	259.06 181.81	0.13 0.06	0.99 0.99	0.12 0.22	0.51 0.61	23.6 21.05	0.92 0.97

### Thermodynamics studies

**Fig 13:**
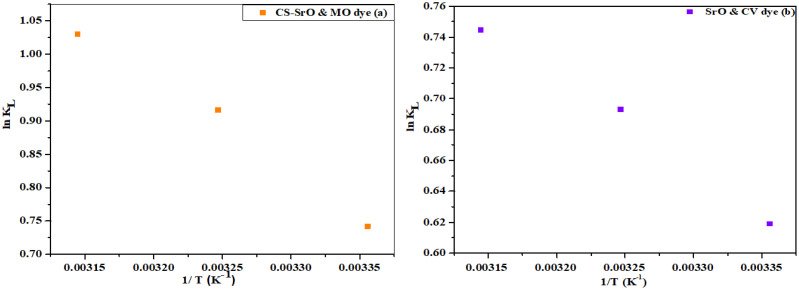
Thermodynamic parameters of dye adsorption (A) MO on CS-SrO nanocomposite (B) CV on SrO nanoparticle.


Various thermodynamic factors such as standard enthalpy ($\Delta H^{\circ }$), standard entropy (ΔS∘) and standard Gibbs free energy (ΔG∘) were calculated to evaluate the temperature impact on adsorption of MO and CV dye by CS-SrO nanocomposite and SrO NPs. These parameters were studied by varying the temperature conditions (298.5, 308.5 and 318.5 K). Values of (ΔS∘), (ΔG∘), and (ΔH∘) for adsorption of MO and CV onto CS-SrO composite and SrO nanoparticles were estimated from the intercept and slope of the plotted curves shown in Fig 13 by applying the following equations:

$$\Delta G=-RTlnK_{c}$$ (7)

$$lnK=\frac{\Delta H\circ }{RT\;}+\frac{\Delta S\circ }{RT\;}\;$$ (8)

$K_{c}$ value is calculated from the given equation:


$$Kc=\frac{qe}{Ce}$$ (9)

Where C_e_ represents the adsorbed dye concentration at equilibrium and q_e_ represents the adsorbate concentration at equilibrium in solution. In both cases, an increase in adsorption was observed at higher temperature conditions as depicted by a straight line graph. This indicates that adsorption is favored at higher temperature values. The negative value of ΔG∘ in both cases show that the adsorption process is spontaneous and feasible at various temperatures. The positive value of ΔH∘ confirms that the reaction for adsorption of MO dye and CV dye on CS-SrO nanocomposite and SrO NPs is endothermic, as presented in Table 3. Moreover, a positive value of ΔS∘ reveals that there is a randomness on the surface of fabricated nanomaterial during the process of adsorption.

**Table 3:** Thermodynamic parameters for adsorption of MO and CV on CS-SrO nanocomposite and SrO nanoparticles respectively.

Material	Dye	T/K	$\Delta G^{\circ }\;(KJ)$	$\Delta H^{\circ }$	$\Delta S^{\circ }\;(KJ)$
**CS-SrO** **nanocomposite**	**MO**	298.5	−1.84	11.35	44.33
		308.5	−2.35		
		318.5	−2.72		
**SrO nanoparticles**	**CV**	298.5	−1.53	4.95	21.77
		308.5	−1.77		
		318.5	−1.97		

### Possible mechanism for SrO nanoparticles formation

**Fig 14:**
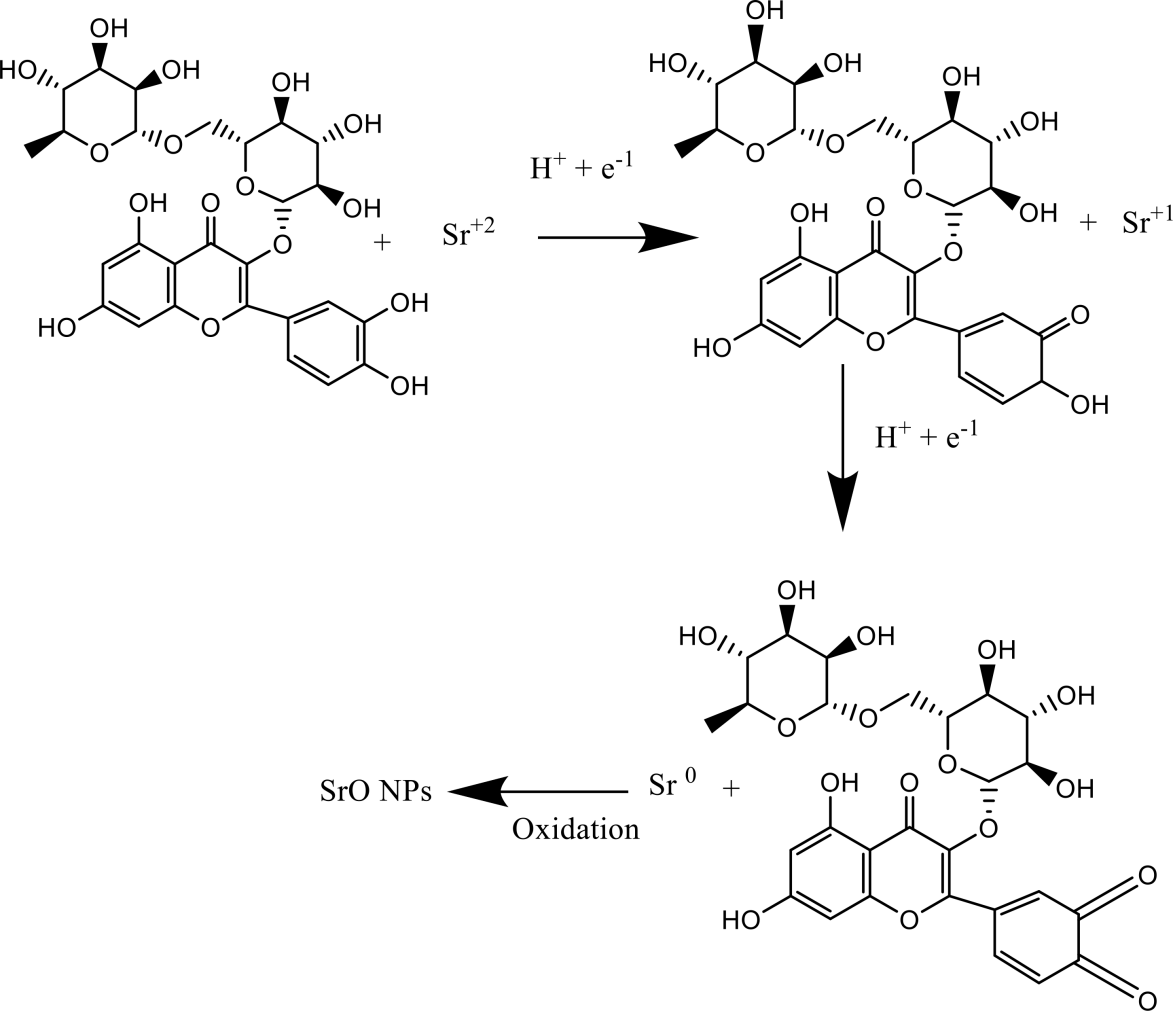
Possible mechanism for reduction of Sr(NO_3_)_2_ to SrO nanoparticles by the phytoconsituents present in Melia azedarach.

In an aqueous leaf extract of *Melia azedarach,* rutin (Quercetin 3-rutino-7-glucoside) act as a reducing agent and is oxidized by strontium nitrate Fig 14. One rutin (plant extract) molecule goes through an O-H bond fission reaction to form a hydrogen radical, which is then transformed into an electron and a proton. Sr^+2^ is reduced to Sr^+1^ ions by the first electron. Sr^+1^ ions are reduced into Sr^0^ in the next step by the fission reaction of the O-H functional group, which creates another electron. Afterward, metal ions are immediately oxidized by atmospheric oxygen to form SrO NPs, which stabilize nanoparticles against the dipole–dipole force.

### Possible mechanism for CS-SrO nanocomposite formation

**Fig 15:**
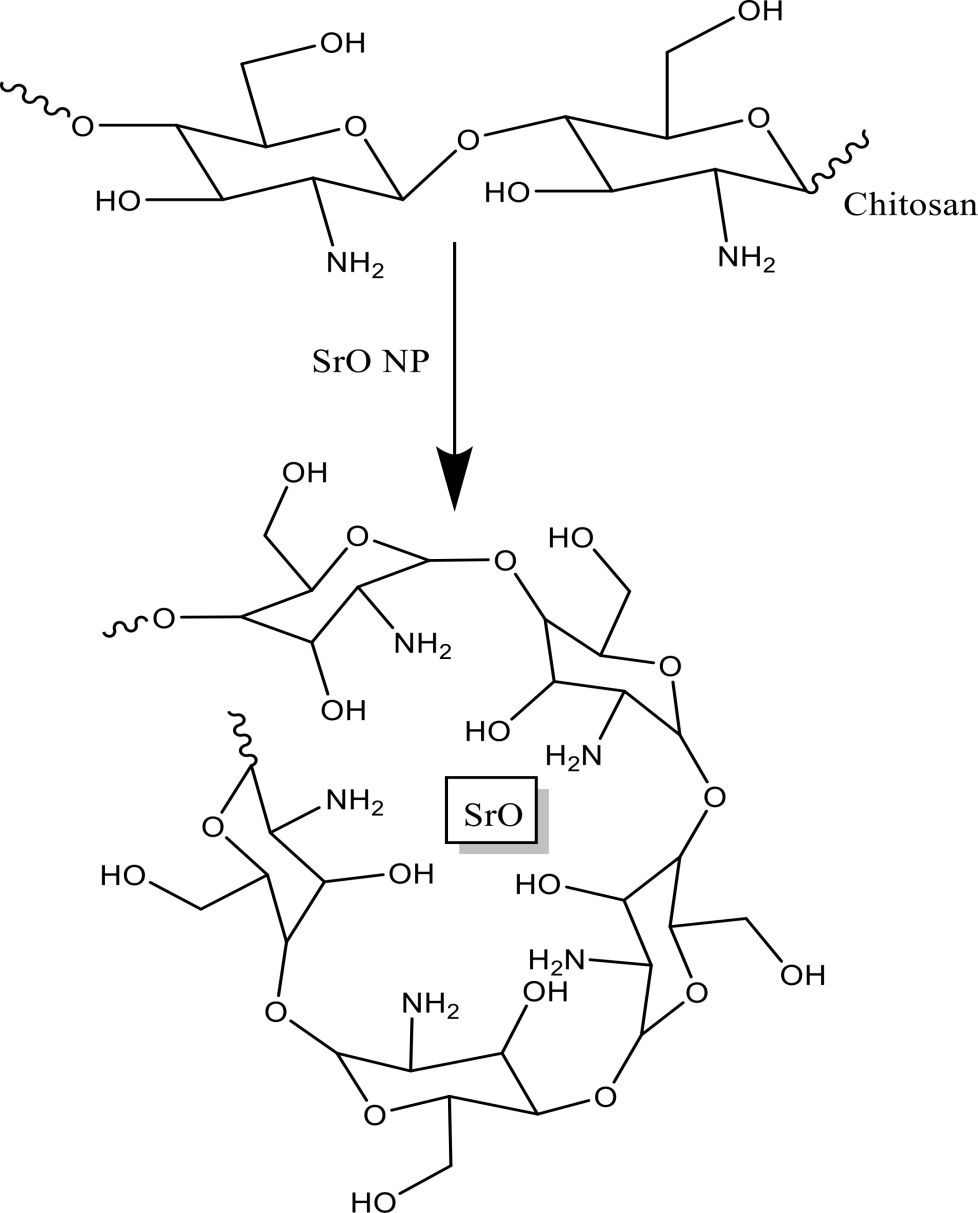
Possible mechanism for formation of CS-SrO nanocomposite.

In this process Fig 15, CS and SrO NPs were mixed in an acetic acid solution, where SrO disintegrated and transformed into strontium cations (Sr^2+^). Coordination bonds were instantly created by the Sr^2+^ ions with -OH and -NH_2_ groups of chitosan. Dropwise addition of NaOH raised the solution’s pH to 5. OH^-^ ions linked up with Sr^2+^ and facilitate the production of the precipitate CS-Sr-OH^+^. The resulting precipitate was ultrasonicated for 4 hours, where the formation of CS-SrO composite was aided by the uniform dispersion of Sr^2+^ in CS solution.

## Conclusion

In this study, strontium oxide NPs and chitosan-strontium oxide nanocomposites were green synthesized and employed for the removal studies of methyl orange (anionic dye) and crystal violet (cationic dye). SrO nanoparticles were found to be more selective for cationic dye (CV) than anionic dye (MO), which may be due to the negatively charged surface of SrO NPs. On the other hand, the CS-SrO nanocomposite showed better results in the case of anionic dye. Adsorption isotherms and thermodynamic studies depicted a feasible, spontaneous and endothermic monolayer adsorption process in both cases. Regeneration studies highlight the reusability factor of prepared materials. Thus, it can be concluded that the synthesis of SrO and CS-SrO using the extract of *Melia azedarach* could be an effective approach for remediation purposes. In terms of future perspectives, these fabricated materials can be exploited for the removal studies of other environmental pollutants

## Abbreviations

NPs: Nanoparticles
SrO: Strontium oxide nanoparticles
CS-SrO: Chitosan-encapsulated strontium oxide nanocomposite
LSPR: Localized surface plasmon resonance
XRD: X-ray diffraction
EDX: Energy-dispersive X-ray spectroscopy
pHPZC: Point of zero charge
CV: Crystal violet (dye)
MO: Methyl orange (dye)
AOP: Advanced oxidation processes
FTIR: Fourier-transform infrared spectroscopy
SEM: Scanning electron microscopy
OA: Oxalic acid
AA: Ascorbic acid
IP: Isopropanol
RL: Langmuir separation factor
ΔG°: Standard Gibbs free energy
ΔH°: Standard enthalpy
ΔS°: Standard entropy
Kc: Equilibrium constant
