# Correction: Catalytic activity evaluation of phyto synthesized SrO and chitosan encapsulated SrO nanomaterials

**DOI:** 10.1371/journal.pone.0349891

**Published:** 2026-05-26

**Authors:** Maria Zaib, Kaif Akram, Tayyaba Shahzadi, Indra Neel Pulidindi, Awais Khalid, Mousa M. Hossin, Hanadi A. Almukhlifi, Ayesha Siddiqua, M. Yasmin Begum

The Funding statement for this article is incorrect. The correct Funding statement is as follows: This research work is funded by the Deanship of Research and Graduate Studies at King Khalid University, Saudi Arabia, through the large Group Research Project under the grant number RGP2/20/46.

There is an error in affiliation 1 for author Maria Zaib. The correct affiliation 1 is: Department of Chemistry, Faculty of Science, Al-Baha University, Al-Baha, Saudi Arabia.
